# Out-of-Pocket Spending for Biologic Drugs After Biosimilar Competition for Medicare Patients

**DOI:** 10.1001/jamanetworkopen.2025.54235

**Published:** 2026-01-15

**Authors:** Jacob S. Riegler, Aaron S. Kesselheim, Benjamin N. Rome

**Affiliations:** 1 Program On Regulation, Therapeutics, And Law, Division of Pharmacoepidemiology and Pharmacoeconomics, Department of Medicine, Brigham and Women’s Hospital and Harvard Medical School, Boston, Massachusetts; 2 Department of Medicine, Cambridge Health Alliance, Cambridge, Massachusetts; 3 Harvard Business School, Boston, Massachusetts

## Abstract

**Importance:**

Biosimilar competition has been associated with in lower prices and decreased US health care spending, but this has not consistently led to lower out-of-pocket (OOP) costs for commercially insured patients using these medications.

**Objective:**

To investigate whether biosimilar competition was associated with lower OOP spending for Medicare patients who used biologics.

**Design, Setting, and Participants:**

This cross-sectional study used an event study design. Participants were patients with Medicare Advantage coverage in a national commercial claims database (Optum’s deidentified Clinformatics Data Mart Database) who used 1 of 7 clinician-administered biologics (filgrastim, infliximab, pegfilgrastim, epoetin alfa, bevacizumab, rituximab, and trastuzumab) from 2009 to 2022. Data were analyzed from April to November 2025.

**Exposure:**

The calendar year when the first biosimilars entered the market for each drug.

**Main Outcomes and Measures:**

The primary outcome was annual OOP spending for all doses of the biologic that a patient received in each year. Two-part regression models were used to estimate mean annual OOP spending in each year, adjusted for patient age, sex, US Census region, diagnosis, and place of service. Results were stratified by whether patients paid coinsurance or deductibles vs those who only paid copayments.

**Results:**

A total of 273 774 patient-years for the 7 drugs were analyzed. Patients had a mean (SD) age of 76 (8) years, 57.6% were female, 53.4% used biologics to treat cancer, 49.7% received biologics in outpatient clinics, and 13.0% received biosimilars. Overall, 52.2% of patients paid coinsurance or deductibles, 5.2% paid only copayments, and 40.8% paid no OOP costs. Across the drugs in the cohort, mean annual OOP costs decreased by $94 (95% CI, −$105 to −$84) after competition, from $233 (95% CI, $228 to $237) in the year before biosimilar competition to $165 (95% CI, $158 to $172) 4 years after competition. Annual OOP costs decreased for all 7 drugs, and decreases were larger among patients who paid coinsurance or deductibles, compared with those who only paid copayments.

**Conclusions and Relevance:**

This cross-sectional study found that biosimilar competition was associated with lower OOP spending for Medicare patients, likely because many patients paid a percentage of drug costs. These findings suggest that by reducing OOP spending, biosimilar competition can improve access and adherence to biologic medications for Medicare patients.

## Key Points

**Question:**

For 7 biologic medications that faced biosimilar competition from 2009-2022, was this competition associated with lower out-of-pocket (OOP) spending for Medicare patients who used these medications?

**Findings:**

In this cross-sectional study including 273 774 patient-years, mean annual OOP spending 4 years after biosimilar competition began decreased by a mean of $94 compared with the year prior to competition.

**Meaning:**

These findings suggest that by reducing OOP spending, biosimilar competition may improve access to biologic medications for Medicare patients.

## Teaser

This cross-sectional study investigates whether biosimilar drug competition was associated with lower out-of-pocket spending for a national sample of Medicare patients who used biologics.

## Introduction

Biologics—complex medications derived from living organisms—treat many different conditions and are a major source of spending in the US.^1^ While biologics represent a small share of prescription drugs used in the US, they accounted for nearly half of US prescription drug spending in 2021.^2^ This disproportionate spending is driven by high prices, which are made possible because patent protection allows biologic drug makers to set monopoly prices.^3,4,5^ To address increasing spending on biologics, in 2009 Congress authorized an expedited US Food and Drug Administration (FDA) pathway to facilitate competition by biosimilars, which are comparable alternatives to original biologics made by different manufacturers.^6^ The goal was for biosimilar competition to lower costs for patients and the health care system, like generic competition has done for small-molecule drugs.

Early biosimilars were priced 15% to 45% lower than the originator biologic.^2,7^ In some cases, this competition also led to lower prices for the originator biolgoics.^8,9^ Overall, biosimilars have been estimated to save the US health care system $56 billion since 2015,^10^ despite the fact that biosimilar uptake after introduction to the US market averages only 25%, compared with 85% or higher uptake of generic drugs.^11,12^

What is less clear, however, is whether lower prices for biosimilars have translated to improved access and lower out-of-pocket (OOP) costs for patients who rely on biologic medications. A 2024 study^3^ found that among commercially-insured patients, OOP costs did not consistently decrease after biosimilar competition began for 7 biologic drugs.^3^ These first biologics to face biosimilar competition were injection or infusion products administered by clinicians in an office or hospital setting; reimbursement by commercial insurers for these drugs varies and can be 3 to 4 times higher than the price of the medication.^13^ Thus, even if competition resulted in lower prices for these drugs, the insurer reimbursements that serve as the basis for patient OOP costs may have been unaffected.^14^

By contrast, Medicare reimburses hospitals and clinics based on average sales prices at which manufacturers sell the drugs to these clinics, inclusive of rebates and discounts.^13^ As a result, it is possible that lower average sales prices from biosimilar competition could directly translate to lower OOP spending for those with Medicare. Understanding whether biosimilar competition lowers OOP spending for patients with Medicare may offer insights into whether competition has improved access to biologic medications, which could lead to improved adherence and patient outcomes.^15,16,17^ In this study, we examined how OOP costs for patients with Medicare Advantage coverage changed after 7 clinician-administered biologics faced biosimilar competition.

## Methods

### Study Design and Data Sources

In this cross-sectional study with an event study design, we used data from Optum’s deidentified Clinformatics Data Mart Database, a national commercial claims database of millions of individuals that includes a large Medicare Advantage population. The study included data from 2009 through 2022. This study was approved by the Massachusetts General Brigham institutional review board with a waiver of informed consent because the use of deidentified data involved no more than minimal risk. We followed the Strengthening the Reporting of Observational Studies in Epidemiology (STROBE) reporting guideline.

### Cohort

We included patients with Medicare Advantage coverage who used at least 1 of 7 clinician-administered biologics that had biosimilar versions available in the US by the end of 2020: filgrastim, infliximab, epoetin alfa, pegfilgrastim, bevacizumab, rituximab, and trastuzumab. Patients with Medicare low-income subsidies and those dually eligible for Medicare and Medicaid were excluded because OOP costs for these patients are substantially lower than other Medicare patients. We excluded bevacizumab users with ophthalmologic diagnosis codes because intravitreal injections require a repackaged lower dose of bevacizumab that was only available for the brand-name version during the study period.^18^

### Exposure

The primary exposure was the calendar year in which biosimilars became available for each drug, which ranged from 2013 for filgrastim to 2019 for bevacizumab, rituximab, and trastuzumab. As a result, each drug had at least 4 years of data before and 3 years after biosimilar competition began.

### Outcomes

The primary outcome was annual OOP spending, defined as the sum of deductibles, copayments, and coinsurance for all doses of the biologic or drug that a patient received in each calendar year, including brand-name and biosimilar versions. This was done to account for the fact that some patients switch between the original and a biosimilar version within a year and because the goal of this study was to assess spending experienced by patients who used any version of the drug rather than evaluate differences in OOP spending by product.

### Covariates

For each patient-year, we measured characteristics including age, sex, US Census region, site of service (outpatient hospital, office, or other), and therapeutic category based on primary diagnosis code associated with the first biologic or biosimilar claim during the calendar year (hematologic, oncologic, rheumatologic, renal, gastrointestinal, ophthalmologic, neurologic, and other or unknown) (eTable 1 in Supplement 1).

### Statistical Analysis

Because spending data were zero-inflated and right-skewed, we used 2-part regression models to estimate mean annual OOP spending per patient. The first part of these models was logit and the second was a generalized linear model with γ distribution with a log link. We reported overall mean results and results from the first part of the model using adjusted odds ratios (aORs) that patients had any nonzero OOP costs. We created separate models for patients who used each drug plus a composite model that included patients who used all drugs with random effects for individual drugs to estimate overall means. All models included fixed effects for patient characteristics and calendar year. Models were analyzed for all users (biosimilar and originator). We modeled mean estimated annual OOP costs and used Wald tests to compare changes in the marginal mean annual spending for each respective year compared to the year before biosimilar market entry. In a sensitivity analysis, we used quantile regression models to assess variation across the annual OOP spending distribution (10th, 25th, 50th, 75th, and 90th percentiles) for patients with nonzero spending.

Patient cost-sharing requirements vary depending on their insurance plan’s design; for example, those patients with deductibles (full cost up to a threshold) and coinsurance (percentage of costs) may be more sensitive to changes in drug prices than those with copayments (flat fees).^19^ To measure how these differences affected trends in OOP spending, we repeated the analysis among 2 subgroups of patients: those who paid any coinsurance or deductibles and those who paid copayments but no coinsurance or deductibles; patients with zero OOP costs were excluded from the subgroup analyses.

For context, we analyzed trends in Medicare payment limits for each of the 7 drugs up to 4 years before and after biosimilar competition began. Payment limits were obtained from public Medicare Average Sales Price files^20^; the limits are reported quarterly based on manufacturer-reported average sales prices.

Analyses were performed using Stata software version 18MP (StataCorp); 2-part models were generated using the twopm package.^21^ Two-sided tests were deemed significant at 2-sided *P* < .05. Data were analyzed from April to November 2025.

## Results

The study included 273 774 patient-years; the mean (SD) patient age was 76 (8) years, 157 630 (57.6%) were female, 146 280 (53.4%) used biologics as part of cancer care, 136 033 (49.7%) received biologics in outpatient clinics, and 35 738 (13.0%) received biosimilars (Table). Pegfilgrastim (78 715 patient-years [28.8%]) and epoetin alfa (63 788 patient-years [23.3%]) accounted for the largest proportion of patient-years, and trastuzumab (12 886 patient-years [4.7%]) accounted for the smallest.

**Table.** Patient Demographics by Patient-Years

Characteristic	Patient-years, No. (%) (N = 273 774)
Age, y	
18-44	1025 (0.4)
45-64	21 796 (8.0)
65-74	127 979 (46.7)
75-80	66 516 (24.3)
>80	56 456 (20.6)
Sex	
Female	157 630 (57.6)
Male	116 144 (42.4)
Geographic region	
Northeast	38 885 (14.2)
Midwest	69 441 (25.4)
South	115 905 (42.34)
West	49 472 (18.1)
Unknown or other	71 (<0.1)
Site of service	
Clinic or office	136 033 (49.7)
Outpatient hospital	95 711 (35.0)
Other^a^	42 030 (15.3)
Biologic (date of biosimilar competition)	
Epoetin alfa (November 2018)	63 788 (23.3)
Filgrastim (November 2013)^b^	24 633 (9.0)
Infliximab (November 2016)	21 933 (8.0)
Rituximab (November 2019)	47 503 (17.4)
Trastuzumab (July 2019)	12 886 (4.7)
Bevacizumab (July 2019)^c^	24 316 (8.9)
Pegfilgrastim (July 2018)	78 715 (28.8)
Received biosimilar version	
No	238 036 (87.0)
Yes^d^	35 738 (13.0)
Associated diagnosis category	
Gastroenterology	6418 (2.3)
Hematology	28 497 (10.4)
Nephrology	21 495 (7.9)
Neurology	1431 (0.5)
Oncology	146 280 (53.4)
Ophthalmology	4131 (1.5)
Rheumatology	25 280 (9.2)
Other/not listed	40 242 (14.7)

^a^ Other sites of service include home, hospice, skilled nursing facilities, and dialysis centers.

^b^ The first filgrastim follow-on product, tbo-filgrastim, was not technically approved through the US Food and Drug Administration’s abbreviated biosimilar approval pathway. Given that tbo-filgrastim was made by a competing manufacturer and approved for similar use as the brand-name medication, tbo-filgrastim was included in the study.

^c^ Patients who used bevacizumab for ophthalmologic diagnoses were excluded because intravitreal injections require use of a repackaged lower-dose version that was only available for the brand-name drug during the study period.

^d^ Includes any patient who received a biosimilar version at least once during the year. This included 30 520 patients (11.1%) who received only biosimilar versions and 5218 patients (1.9%) who received both brand-name and biosimilar versions during the year.

### Annual OOP Spending

**Figure 1.**
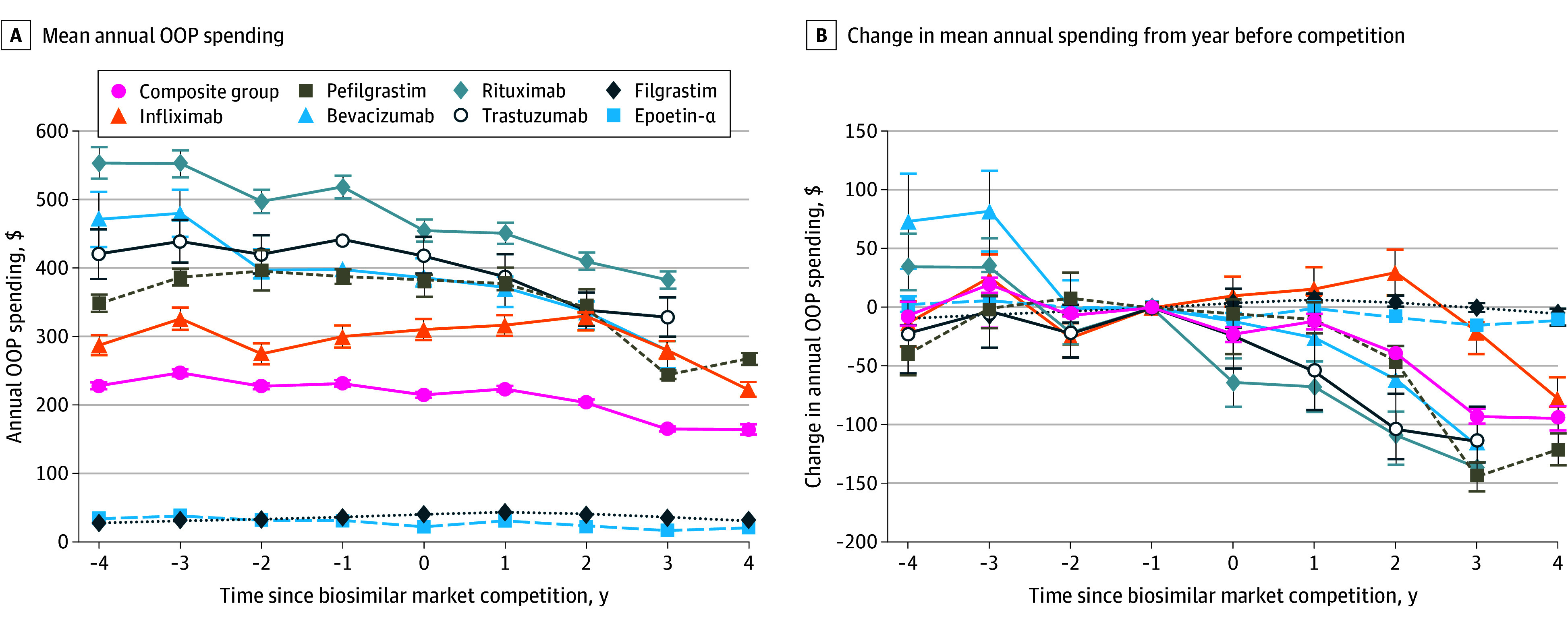
Mean Out-of-Pocket (OOP) Spending and Changes in Spending Before and After Biosimilar Competition Error bars represent 95% CIs.

In the year before biosimilar competition, mean annual OOP spending ranged from $32 (95% CI ,$29 to $34) for epoetin alfa to $520 (95% CI, $503 to $536) for rituximab. For 3 drugs (epoetin alfa, bevacizumab, rituximab), mean OOP costs were decreasing before biosimilar competition; for the 4 other drugs, mean annual OOP costs were stable or rising before competition. Mean annual OOP spending decreased after biosimilar competition began for all 7 drugs, with the smallest decrease observed for filgrastim (−$5 [95% CI, −$10 to −$1) and the largest for rituximab (−$136 [95% CI, −$159 to −$115]) (Figure 1; eFigure 1 in Supplement 1).

**Figure 2.**
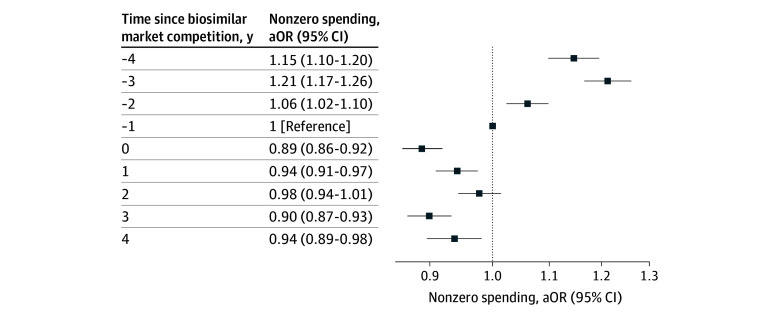
Adjusted Odds Ratios (aORs) of Nonzero Out-of-Pocket Spending Before and After Biosimilar Competition Results are the mean across the 7 biologics; results for individual drugs are shown in eTable 2 in Supplement 1.

In the composite model including all users, mean annual OOP costs decreased by $94 (95% CI, −$105 to −$84) after competition, from $233 (95% CI, $228 to $237) in the year before biosimilar competition (year −1) to $165 (95% CI, $158 to $172) 4 years after competition. The proportion of patients with zero OOP spending remained relatively similar over time, with 14 051 of 34 007 patient-years (41.3%) in the year prior to biosimilar entry and 7253 of 17 610 patient-years (41.2%) 4 years after market entry (eFigure 2 in Supplement 1). In the adjusted model including all drugs, there was a modestly smaller odds of patients having nonzero OOP spending 4 after biosimilar competition compared to the year prior competition (aOR, 0.94 [95% CI 0.89 to 0.98]), with variation by drug (Figure 2; eTable 2 in Supplement 1).

**Figure 3.**
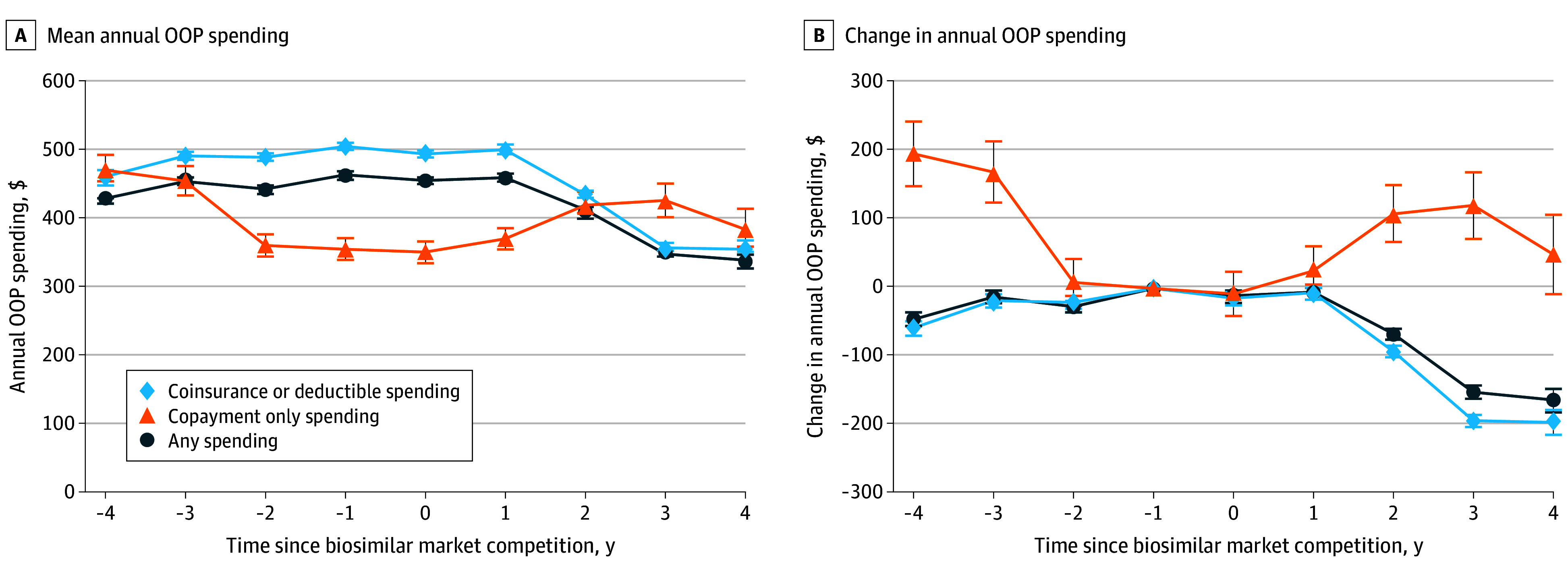
Mean Out-of-Pocket (OOP) Spending and Changes in Spending Before and After Biosimilar Competition by Medicare Plan Type Error bars represent 95% CIs.

Among patients with any nonzero costs, mean OOP costs decreased by $163 (95% CI, −$181 to −$148) after competition, from $465 (95% CI, $460 to 470) to $341 (95% CI, $329 to $352) (Figure 3). Overall, 142 846 patient-years (52.2%) had OOP costs, including any coinsurance or deductible spending, and 19 254 patient-years (5.2%) had copayment spending only. Among patient-years with coinsurance or deductible costs, 141 749 (99.2%) had coinsurance spending and 1097 (0.8%) had deductible spending.

Among patient-years with coinsurance or deductibles, mean annual OOP spending decreased by $197 (95% CI, −$215 to −$179), from $506 (95% CI, $501 to $512) before biosimilar competition to $356 (95% CI, $344 to $369) 4 years after competition for the composite group (Figure 3). All drugs except filgrastim decreased in spending (−$2 [95% CI, −$11 to $8]); the largest reduction was seen for rituximab (−$394 [95% CI, −$418 to −$370]) (eFigure 3 in Supplement 1). By contrast, among patients with copayment spending only, there was no change in OOP costs 4 years after biosimilar market competition compared with the year prior to competition (mean change, $49 [95% CI, −$9 to $58]) (Figure 3). This varied by drug, with increasing OOP costs for some and decreasing costs for others (eFigure 4 in Supplement 1).

### Sensitivity Analysis

**Figure 4.**
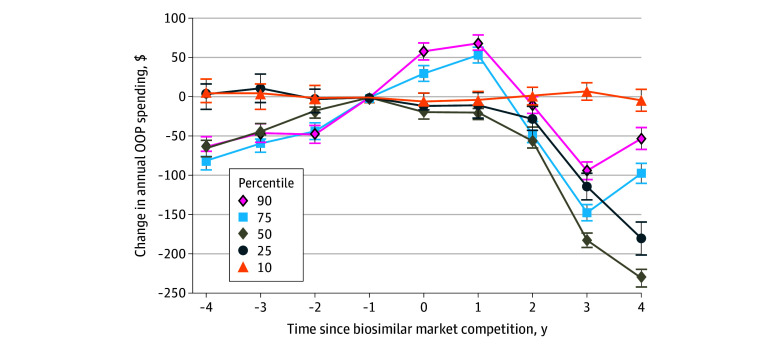
Changes in Out-of-Pocket (OOP) Spending Before and After Biosimilar Competition by Annual Spending Percentile Results are the mean across the 7 biologics; results for individual drugs are shown in eTable 3 in Supplement 1.

In a quantile regression model including all 7 drugs, median OOP spending in the year before competition ranged from $92 (95% CI, $84 to $100) in the 10th percentile to $949 (95% CI, $941 to $956) in the 90th percentile (Figure 4). Four years after biosimilar competition, spending ranged from $89 (95% CI, $77 to $100) in the 10th percentile to $896 (95% CI, $885 to $908) in the 90th (eTable 3 in Supplement 1). Overall, the median reduction in annual OOP spending was $231 (95% CI, −$242 to −$219). Except those in the 10th percentile, all others experienced a reduction in OOP costs relative to the year before biosimilar market competition. The distribution of spending was skewed toward higher spending patients (eTable 3 in Supplement 1). Results for individual drugs are shown in eFigure 5 in Supplement 1.

### Comparison With Average Sales Prices

The average sales prices for all originator drugs, except epoetin alfa, increased before biosimilar market entry and declined thereafter, except for filgrastim, which continued to increase for 1 year before stabilizing. Biosimilar prices were lower than the originator and decreased over time for all 7 drugs (eFigure 6 in Supplement 1).

## Discussion

In this cross-sectional event study of Medicare patients who received 1 of 7 clinician-administered biologic medications, annual OOP costs were substantially lower after these drugs faced biosimilar competition, a finding that was consistent across the drugs included in this study. These findings suggest that patients who used biologic medications directly benefited from biosimilar competition. Lower costs for patients could translate to improved medication adherence and better clinical outcomes.

Biosimilar competition has resulted in a mean 56% reduction in prices and 51% less spending by payers, even when most patients continue to use the brand-name drugs rather than adopting biosimilars.^4,10,22,23^ However, few studies have examined how these translate to OOP spending by patients who use these medications. A 2024 study^3^ found that biosimilar competition was not consistently associated with lower OOP spending for commercially insured patients. Our study found that the experience for patients in Medicare was different, with biosimilar competition associated with lower patient OOP spending.

Two main factors likely contribute to the associations of biosimilar competition for Medicare patients. First, Medicare reimbursement for clinician-administered biologics is directly linked to average sales prices. As in prior studies,^9^ we found that these prices, which are inclusive of most discounts and rebates, decreased after biosimilar competition for 6 of 7 drugs in our study. By contrast, commercial insurers negotiate reimbursements for clinics and hospitals that vary and can be 3 to 4 times more than average sales prices.^13^ Because OOP costs are based on insurer reimbursements in the commercial market, patients may be unable to benefit in the same way from lower prices due to biosimilar competition.

Second, approximately one-half of Medicare patients in our study paid coinsurance or deductibles that are derived from the cost of the drug. Lower OOP costs after biosimilar competition were only observed among this subset of patients. A similar proportion of patients had no OOP costs after competition, and there was no change in OOP costs observed among patients who only paid flat fees (copayments) that are not directly connected to the drug’s price.

So far in the US, biosimilar uptake has remained modest, representing less than 30% of prescriptions when they are available.^11^ Additionally, biosimilar entry is often delayed by thickets of patents on brand-name biologics that limit timely competition.^24^ Our study suggests that these delays and the limited uptake of biosimilars may be handicapping the potential benefits of biosimilar competition for Medicare patients’ access to biologic drugs by maintaining higher OOP costs. High OOP costs have been linked with lower treatment initiation and prescription fill rates,^15^ underscoring the importance of lowering these costs to improve access and adherence to treatment.

### Limitations

This study has several limitations. Claims data do not capture manufacturer patient assistance programs, so actual spending by patients could be overestimated.^19^ Additionally, the analysis was restricted to a single dataset of Medicare Advantage enrollees, which may limit generalizability to other Medicare patients, including those with fee-for-service Medicare, where biologic drugs accounted for an estimated 79% of Part B drug spending in 2021.^25^ There could also have been changes in plan design over time that were independent of biosimilar market entry. For example, Medicare Advantage plans have a ceiling maximum OOP costs for inpatient and outpatient medical care that increased from $6700 in 2011 to 2020 to $7550 in 2021 to 2022; this may have led us to underestimate the impact of biosimilar competition.^26,27^ Prior to 2011, there were no regulatory caps on OOP spending for Medicare Advantage plans.^25,26^ Results from this study are limited to clinician-administered biologics and are not generalizable to pharmacy-administered biologics (eg, adalimumab), as these are covered under Medicare Part D and are reimbursed through separate mechanisms.^28^ For example, starting in 2025, Medicare Part D prescription drug coverage included a $2000 annual OOP cap, which may result in savings for patients who use brand-name biologics or biosimilars.^29^

## Conclusions

The findings from this cross-sectional event study indicate that biosimilar competition was associated with lower OOP spending for Medicare patients. Ensuring robust and timely biosimilar competition and encouraging the use of biosimilars are 2 strategies that could improve affordability and access to biologic drugs for the benefit of Medicare patients.

## Notes

Supplement 1. eTable 1. *ICD-9* and *ICD-10* code prefixes used to group claims by clinical categories eFigure 1. Mean out-of-pocket costs for filgrastim and epoetin and change in out-of-pocket costs relative to the year before biosimilar market entry eFigure 2. Raw percentage of patients with zero out-of-pocket spending for each drug and mean of the 7 drugs each year from biosimilar market entry and competition eTable 2. Adjusted odds ratio of nonzero out-of-pocket spending relative to the year prior to biosimilar market entry for a group of all 7 drugs, as well as each individual biologic eFigure 3. Change in mean predicted out-of-pocket spending for patients with coinsurance or deductible spending compared to the year prior to biosimilar market competition for each drug eFigure 4. Change in mean estimated out-of-pocket spending for patients with copayment only compared to the year prior to biosimilar market competition for each drug eTable 3. Estimated out-of-pocket spending for a composite group consisting of all study biologics by percentiles each year from biosimilar market entry and competition eFigure 5. Change in median estimated out-of-pocket spending compared to the year before biosimilar entry for each drug eFigure 6. Trends in average sales price for originator biologics and their corresponding biosimilars during the study period

Supplement 2. Data Sharing Statement
