# Supplementary material for: Out-of-Pocket Spending for Biologic Drugs After Biosimilar Competition for Medicare Patients
Source: JAMA Netw Open. 2026 Jan 15;9(1):e2554235. doi: 10.1001/jamanetworkopen.2025.54235 (PMC12809370; doi:10.1001/jamanetworkopen.2025.54235)
Supplement: Supplement 1. — eTable 1. ICD-9 and ICD-10 code prefixes used to group claims by clinical categories eFigure 1. Mean out-of-pocket costs for filgrastim and epoetin and change in out-of-pocket costs relative to the year before biosimilar market entry eFigure 2. Raw percentage of patients with zero out-of-pocket spending for each drug and mean of the 7 drugs each year from biosimilar market entry and competition eTable 2. Adjusted odds ratio of nonzero out-of-pocket spending relative to the year prior to biosimilar market entry for a group of all 7 drugs, as well as each individual biologic eFigure 3. Change in mean predicted out-of-pocket spending for patients with coinsurance or deductible spending compared to the year prior to biosimilar market competition for each drug eFigure 4. Change in mean estimated out-of-pocket spending for patients with copayment only compared to the year prior to biosimilar market competition for each drug eTable 3. Estimated out-of-pocket spending for a composite group consisting of all study biologics by percentiles each year from biosimilar market entry and competition eFigure 5. Change in median estimated out-of-pocket spending compared to the year before biosimilar entry for each drug eFigure 6. Trends in average sales price for originator biologics and their corresponding biosimilars during the study period [file jamanetwopen-e2554235-s001.pdf]

Supplemental Online Content

Riegler JS, Kesselheim AS, Rome BN. Out-of-pocket spending for biologic drugs after biosimilar competition for Medicare patients. *JAMA Netw Open*. 2026;9(1):e2554235. doi:10.1001/jamanetworkopen.2025.54235

eTable 1. *ICD-9* and *ICD-10* code prefixes used to group claims by clinical categories

eFigure 1. Mean out-of-pocket costs for filgrastim and epoetin and change in out-of-pocket costs relative to the year before biosimilar market entry

eFigure 2. Raw percentage of patients with zero out-of-pocket spending for each drug and mean of the 7 drugs each year from biosimilar market entry and competition

eTable 2. Adjusted odds ratio of nonzero out-of-pocket spending relative to the year prior to biosimilar market entry for a group of all 7 drugs, as well as each individual biologic

eFigure 3. Change in mean predicted out-of-pocket spending for patients with coinsurance or deductible spending compared to the year prior to biosimilar market competition for each drug

eFigure 4. Change in mean estimated out-of-pocket spending for patients with copayment only compared to the year prior to biosimilar market competition for each drug

eTable 3. Estimated out-of-pocket spending for a composite group consisting of all study biologics by percentiles each year from biosimilar market entry and competition

eFigure 5. Change in median estimated out-of-pocket spending compared to the year before biosimilar entry for each drug

eFigure 6. Trends in average sales price for originator biologics and their corresponding biosimilars during the study period

This supplemental material has been provided by the authors to give readers additional information about their work.

© 2025 Riegler JS et al. *JAMA Network Open*.

eTable 1. *ICD-9* and *ICD-10* code prefixes used to group claims by clinical categories.

Clinical Category	ICD-9 Prefix	ICD-10 Prefix	HCPCS Codes
Hematology	279-289	D50-89	Diseases of the blood and immune system
Oncology	140-239	C, D00-49	Neoplasms
		Z51	Encounters for chemotherapy
Rheumatology	680-709	L	Diseases of the skin and subcutaneous tissue
	710-739	M	Diseases of the musculoskeletal system and connective tissue
	446	I70-79	Diseases of arteries
Nephrology	580-629	N	Diseases of the genitourinary system
Gastroenterology	520-579	K	Diseases of the digestive system
Ophthalmology	250	E	Diabetes mellitus (with ophthalmologic manifestations)
	360-379	H	Diseases of the eye
Neurology	320-359	G	Diseases of the nervous system

© 2025 Riegler JS et al. *JAMA Network Open*.

eFigure 1. Figure A shows the mean out-of-pocket costs for filgrastim and epoetin and Figure B shows the change in out-of-pocket costs relative to the year before biosimilar market entry. Erro bars represent 95% confidence intervals. These were separated to better visualize trends as out-of-pocket costs were relatively small compared to other study biologics.

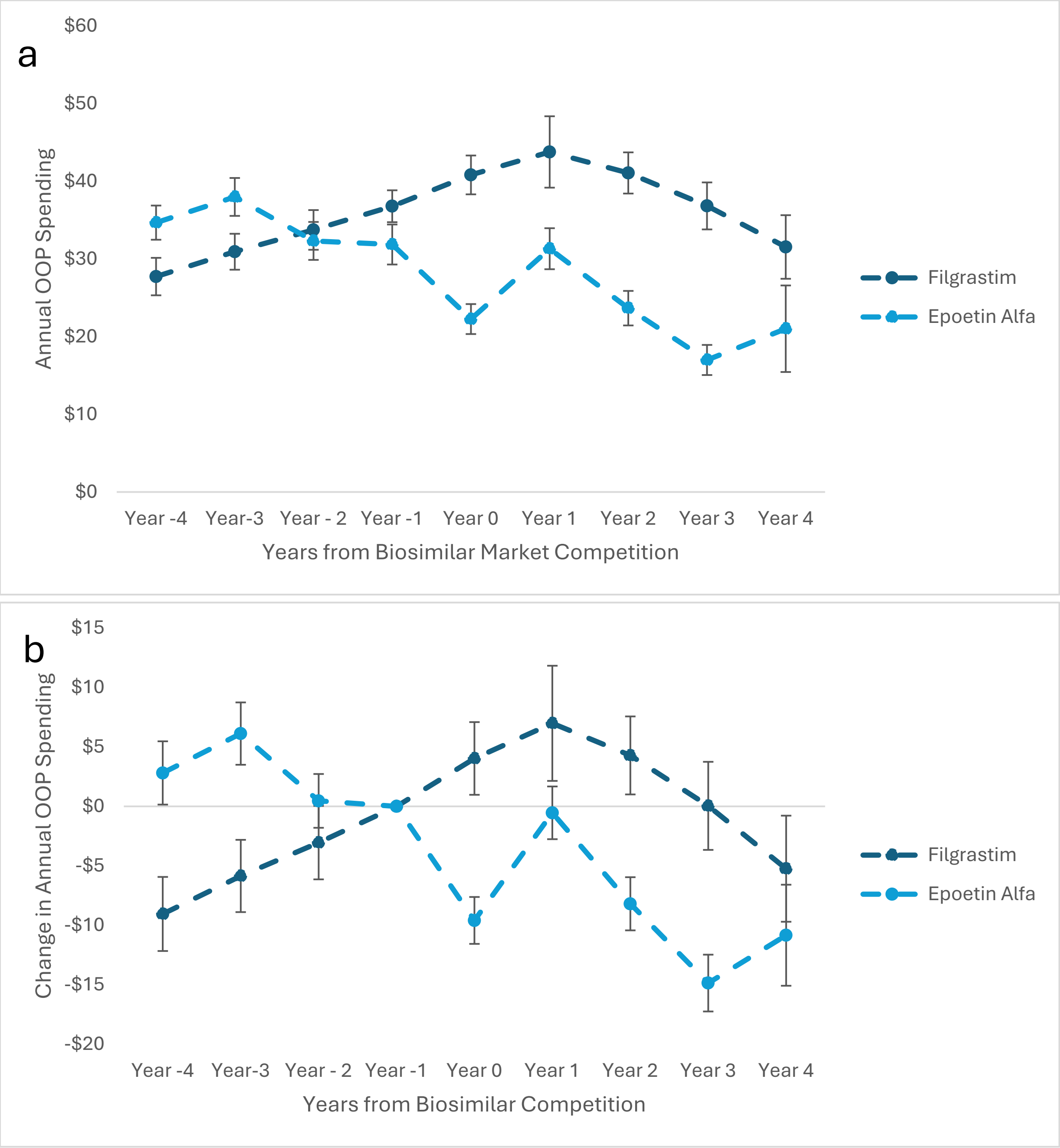

© 2025 Riegler JS et al. *JAMA Network Open*.

eFigure 2. Raw percentage of patients with zero out-of-pocket spending for filgrastim, infliximab, pegfilgrastim, epoetin alfa, bevacizumab, rituximab, trastuzumab, and an average of the 7 drugs (composite group) each year from biosimilar market entry and competition.

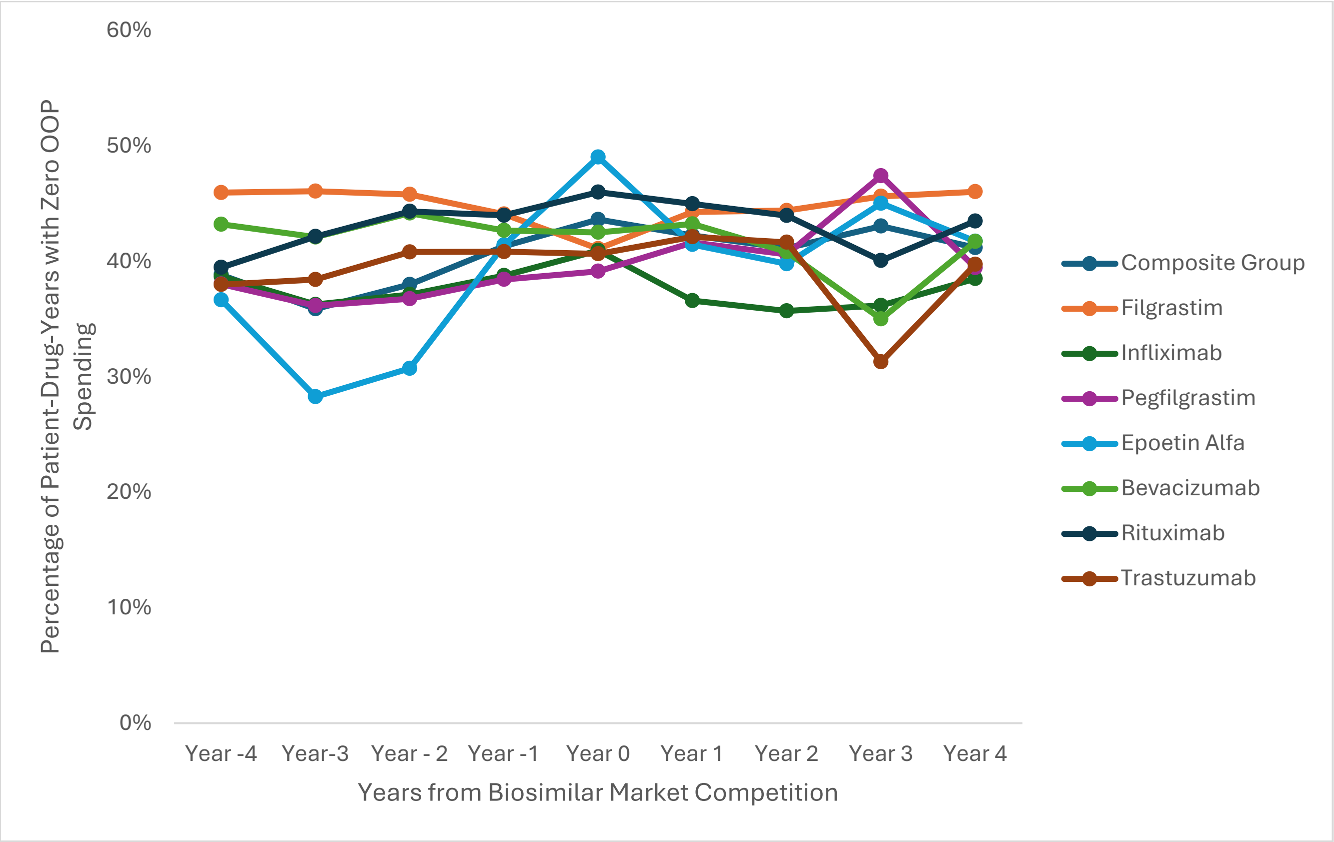

© 2025 Riegler JS et al. *JAMA Network Open*.

eTable 2. Adjusted odds ratio of non-zero out-of-pocket spending relative to the year prior to biosimilar market entry (year -1) for a group of all 7 drugs (composite group) as well as each individual biologic.

Years from biosimilar competition	Composite group AOR (95%CI)	Filgrastim AOR (95%CI)	Infliximab AOR (95% CI)	Pegfilgrastim AOR (95% CI)	Epoetin Alfa AOR (95% CI)	Bevacizumab AOR (95% CI)	Rituximab AOR (95% CI)	Trastuzumab AOR (95% CI)
Year -4	1.15 (1.10–1.20)	0.91 (0.78, 1.07)	1.03 (0.91, 1.16)	1.18 (1.08–1.29)	1.21 (1.09–1.35)	1.10 (0.96–1.25)	1.35 (1.23–1.49)	1.25 (1.06–1.47)
Year -3	1.21 (1.17–1.26)	1.02 (0.88, 1.18)	1.18 (1.05, 1.32)	1.34 (1.23–1.46)	1.40 (1.26–1.56)	1.13 (1.00–1.27)	1.15 (1.06–1.25)	1.17 (1.02–1.33)
Year -2	1.06 (1.02–1.10)	0.99 (0.86, 1.13)	1.15 (1.04, 1.28)	1.15 (1.07–1.24)	1.04 (0.95–1.14)	1.01 (0.91–1.13)	1.03 (0.96–1.10)	1.03 (0.91–1.15)
Year -1	Reference							
Year 0 (biosimilar market entry)	0.89 (0.86–0.92)	1.12 (0.99, 1.27)	0.91 (0.83, 1.00)	0.93 (0.87–1.00)	0.61 (0.55–0.66)	1.04 (0.94–1.15)	0.90 (0.85–0.97)	0.97 (0.87–1.09)
Year 1	0.94 (0.91–0.97)	1.01 (0.89, 1.15)	1.05 (0.94, 1.16)	0.85 (0.80–0.91)	1.06 (0.96–1.16)	0.99 (0.89–1.10)	0.92 (0.86–0.99)	0.88 (0.77–1.01)
Year 2	0.98 (0.94–1.01)	0.99 (0.88, 1.13)	1.04 (0.93, 1.16)	0.92 (0.86–0.98)	0.92 (0.83–1.02)	1.08 (0.98–1.20)	1.02 (0.95–1.10)	0.93 (0.81–1.08)
Year 3	0.90 (0.87–0.93)	0.89 (0.78, 1.02)	1.02 (0.91, 1.15)	0.67 (0.62–0.71)	0.68 (0.61–0.77)	1.40 (1.24–1.57)	1.24 (1.15–1.34)	1.44 (1.22–1.70)
Year 4	0.94 (0.89–0.98)	0.83 (0.73, 0.95)	0.96 (0.85, 1.07)	0.98 (0.91–1.06)	0.67 (0.58–0.78)	—	—	—

© 2025 Riegler JS et al. *JAMA Network Open*.

eFigure 3. Change in mean predicted out-of-pocket spending for patients with coinsurance or deductible spending compared to the year prior to biosimilar market competition (year -1) for (a) infliximab, pegfilgrastim, bevacizumab, rituximab, and trastuzumab (b) filgrastim and epoetin alfa. Graphs were separated for data visualization purposes.

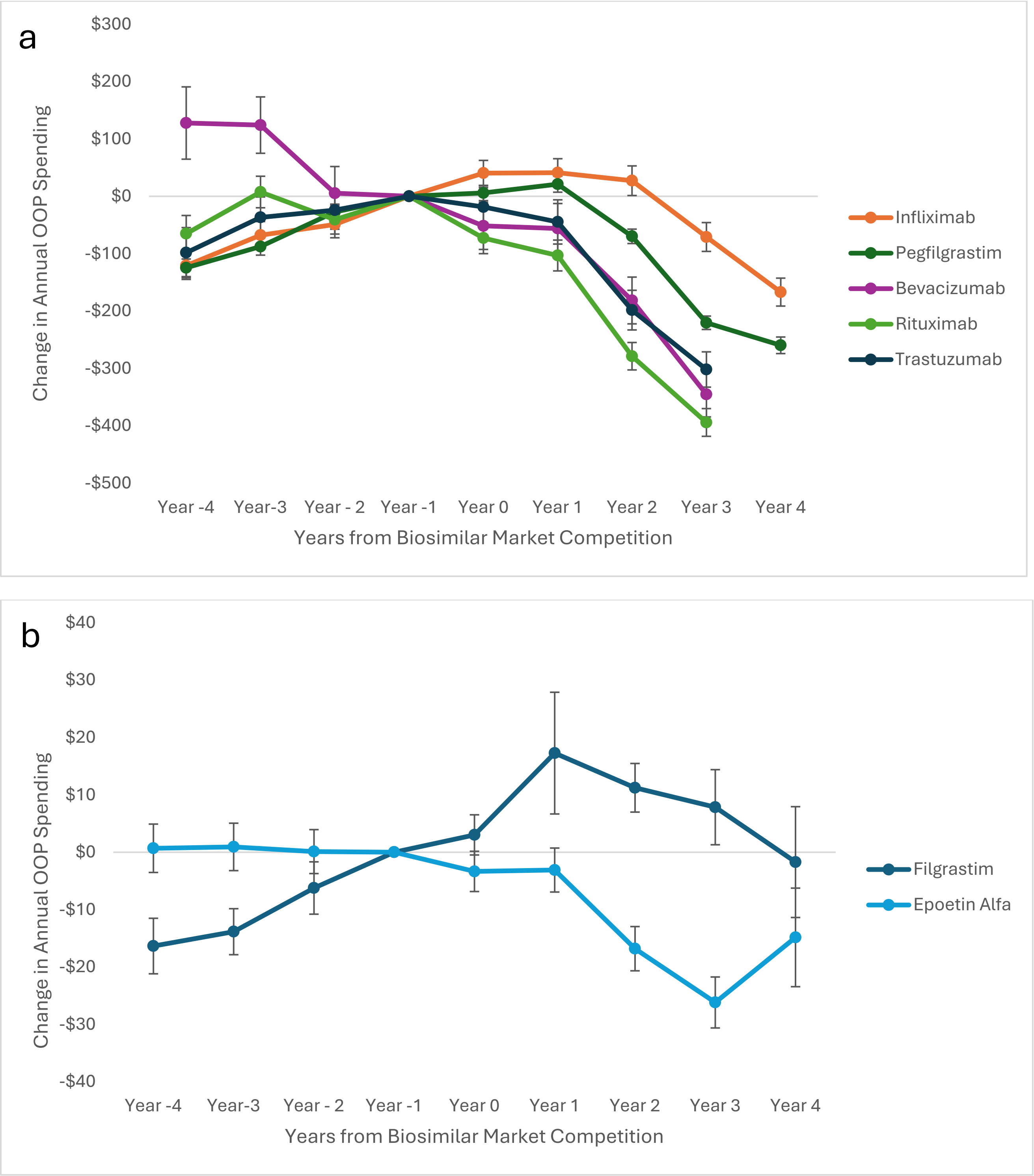

© 2025 Riegler JS et al. *JAMA Network Open*.

eFigure 4. Change in mean predicted out-of-pocket spending for patients with copayment only compared to the year prior to biosimilar market competition (year -1) for (a) pegfilgrastim, bevacizumab, rituximab, and trastuzumab (b) filgrastim, infliximab, and epoetin alfa. Graphs were separated for data visualization purposes.

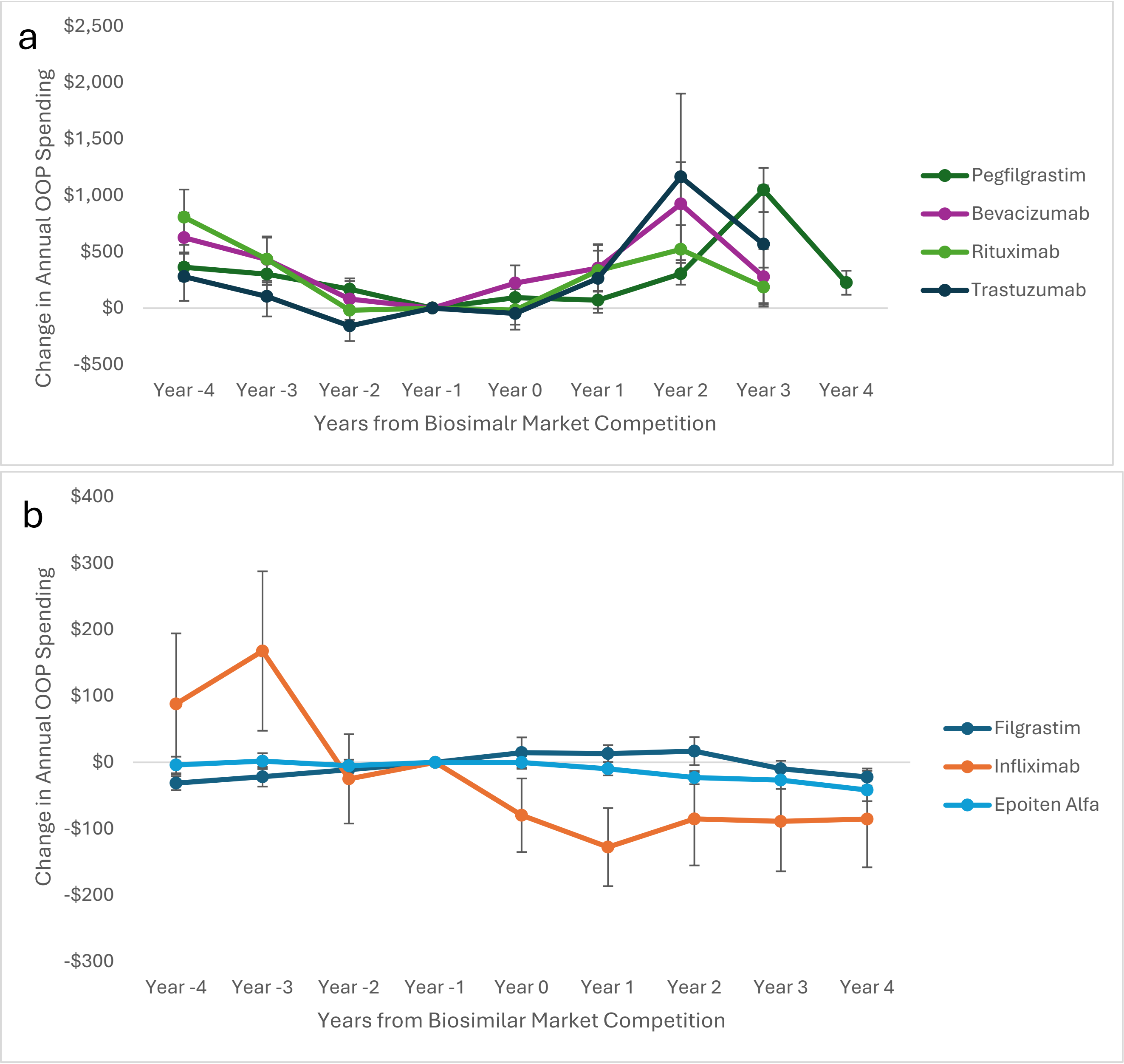

© 2025 Riegler JS et al. *JAMA Network Open*.

eTable 3. Predicted out-of-pocket spending for a composite group consisting of all study biologics across 10th, 25th, 50th, 75th, and 90th percentiles by each year from biosimilar market entry and competition. Analysis was conducted via quantile regression.

Years from Biosimilar Market Introduction	10th percentile costs (95%CI)	25th percentile costs (95%CI)	50th percentile costs (95%CI)	75th percentile costs (95%CI)	90th percentile costs (95%CI)
Year -4	$97 ($87–$108)	$404 ($388–$419)	$564 ($558–$569)	$726 ($717–$736)	$886 ($876–$896)
Year -3	$98 ($88–$107)	$411 ($398–$425)	$585 ($582–$588)	$748 ($740–$757)	$903 ($894–$912)
Year -2	$91 ($83–$100)	$398 ($385–$410)	$611 ($607–$615)	$764 ($756–$772)	$902 ($893–$910)
Year -1	$92 ($84–$100)	$400 ($388–$411)	$628 ($623–$634)	$807 ($800–$814)	$949 ($941–$956)
Year 0 (biosimilar market entry)	$87 ($80–$95)	$389 ($377–$401)	$610 ($606–$613)	$838 ($831–$845)	$1,007 ($1,000–$1,015)
Year 1	$89 ($81–$97)	$390 ($378–$401)	$609 ($604–$614)	$861 ($854–$868)	$1,018 ($1,010–$1,025)
Year 2	$95 ($87–$102)	$372 ($361–$384)	$573 ($569–$576)	$759 ($753–$766)	$939 ($932–$947)
Year 3	$100 ($92–$108)	$286 ($274–$298)	$446 ($440–$452)	$660 ($652–$667)	$855 ($847–$863)
Year 4	$89 ($77–$100)	$220 ($203–$237)	$398 ($391–$405)	$710 ($700–$721)	$896 ($885–$908)

© 2025 Riegler JS et al. *JAMA Network Open*.

eFigure 5. Change in median predicted out-of-pocket spending compared to the year before biosimilar entry (year -1) for (a) infliximab, pegfilgrastim, bevacizumab, rituximab, and trastuzumab (b) filgrastim and epoetin alfa. Graphs separated for data visualization purposes as filgrastim and epoetin alfa had substantially smaller overall out-of-pocket spending.

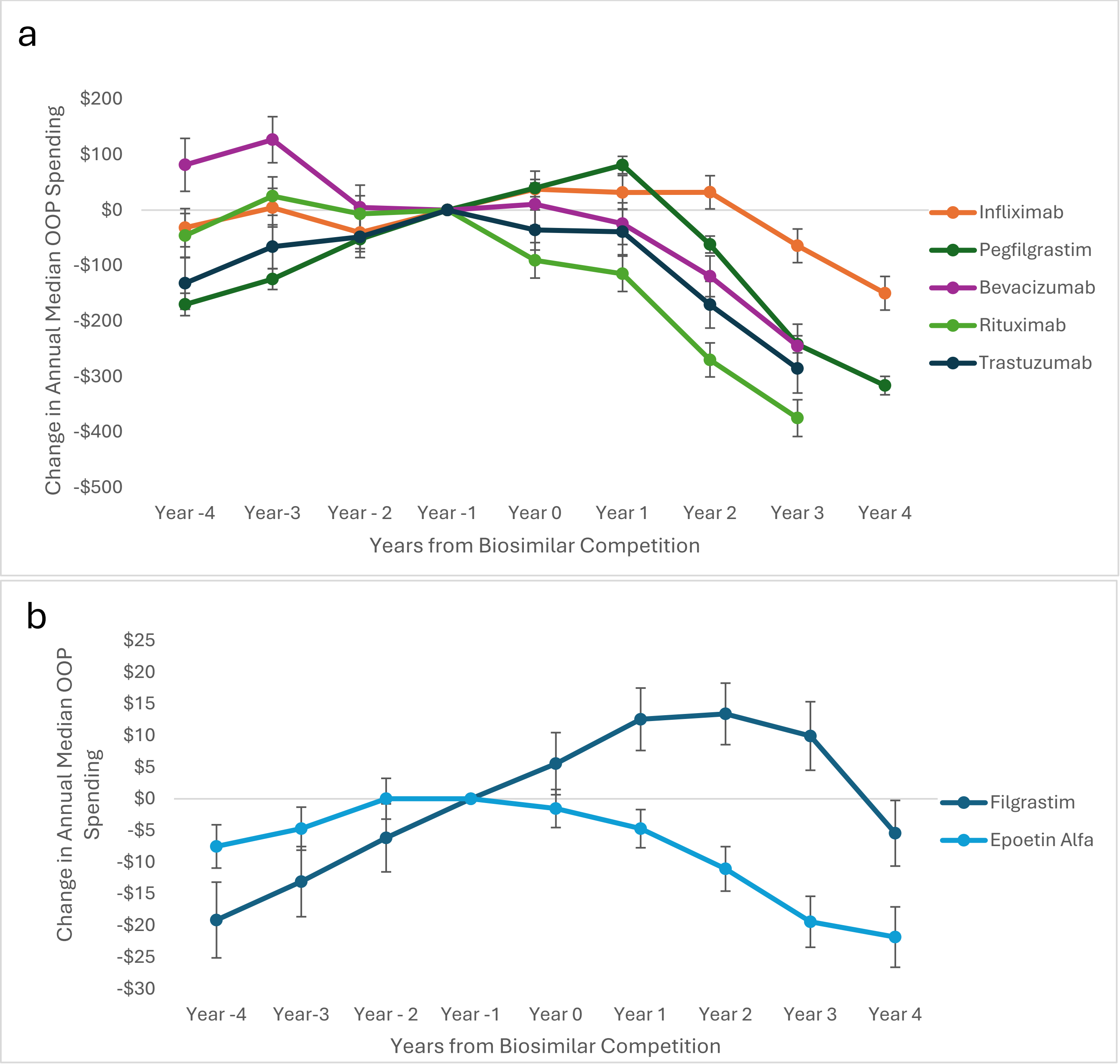

© 2025 Riegler JS et al. *JAMA Network Open*.

eFigure 6. Trends in average sales price (ASP) for originator biologics and their corresponding biosimilars during the study period. Drugs with biosimilar launch dates in November are not shown in the year of entry because quarterly ASP data do not capture partial-year pricing changes.

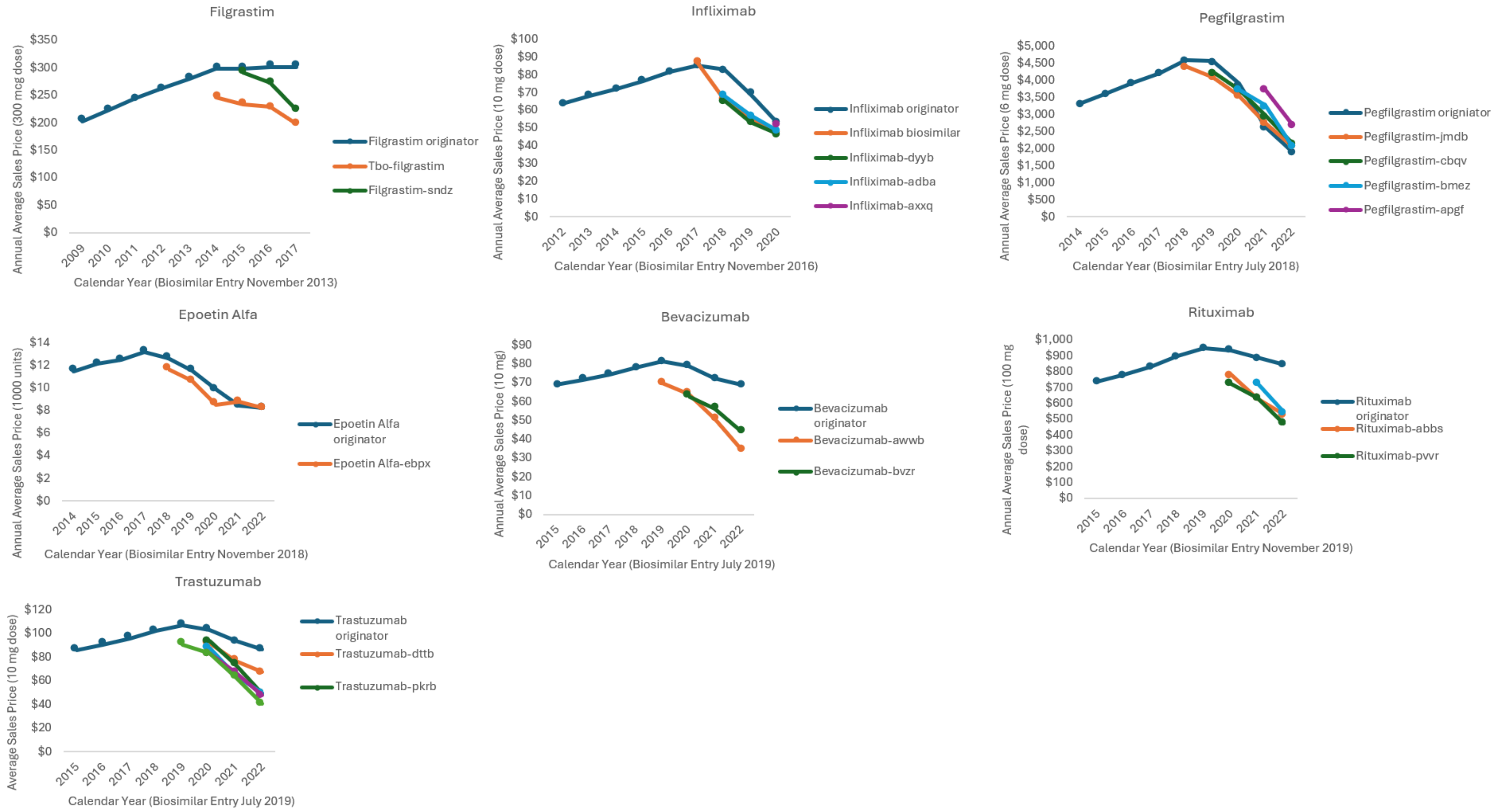

© 2025 Riegler JS et al. *JAMA Network Open*.
