# Supplementary material for: Out-of-Pocket Spending for Biologic Drugs After Biosimilar Competition for Medicare Patients
Source: JAMA Netw Open. 2026 Jan 15;9(1):e2554235. doi: 10.1001/jamanetworkopen.2025.54235 (PMC12809370; doi:10.1001/jamanetworkopen.2025.54235)
Supplement: Supplement 2. — Data Sharing Statement [file jamanetwopen-e2554235-s002.pdf]

Data Sharing Statement

Riegler. Out-of-Pocket Spending for Biologic Drugs After Biosimilar Competition for Medicare Patients. *JAMA Netw Open*. Published January 15, 2026.
doi:10.1001/jamanetworkopen.2025.54235

Data
Data available: No
